# Impact of Nutritional Factors on Length of Hospital Stay and Readmission Risk in a Reference Unit for Eating Disorders

**DOI:** 10.3390/nu18060965

**Published:** 2026-03-18

**Authors:** Carlos Nagore González, Claudia Aparicio Callén, Laura Escartín Madurga, Gloria Bueno Lozano, Gerardo Rodríguez Martínez, Elena Faci Alcalde

**Affiliations:** 1 Department of Pediatrics, Hospital Clínico Universitario Lozano Blesa, 50009 Zaragoza, Spain; 2 Instituto de Investigación Sanitaria Aragón (IISAragón), 50009 Zaragoza, Spain; 3 Department of Pediatrics, University of Zaragoza, 50009 Zaragoza, Spain

**Keywords:** eating disorders, pediatric nutrition, readmission, hospitalization, malnutrition

## Abstract

Introduction: Eating Disorders (ED) represent a significant health concern in the pediatric population due to high morbidity, prolonged hospital stays, and frequent readmissions. Scientific evidence regarding nutritional factors that may influence length of stay or risk of readmission is limited in this population. Objectives: To identify variables associated with longer hospital stays and readmission in pediatric patients with ED admitted to a reference unit in northern Spain. Methods: A retrospective observational study was conducted following STROBE guidelines, including patients under 18 years admitted for ED at a tertiary referral hospital between 2022 and 2025. Nutritional, anthropometric, clinical, evolution-related, and treatment variables were collected. Descriptive analyses, group comparisons according to length of stay and readmission, and logistic regression models were performed to identify associated factors. Results: The study included 75 patients, predominantly female (94.7%), with a mean age of 14.5 years. Twenty-eight percent of patients experienced at least one readmission during the study period. Multivariable regression identified that the use of a nasogastric tube and nutritional supplements was significantly associated with reduced length of stay. In addition, in patients with moderate to severe malnutrition, a recovery greater than 5% according to the Waterlow index was associated with a lower probability of readmission. Although anthropometric differences were observed between groups according to their need for readmission, most were not statistically significant. Conclusions: Nutritional support via nasogastric tube when indicated, the use of nutritional supplements, and a >5% recovery in the Waterlow index in patients with moderate to severe malnutrition are key factors in reducing hospital stay and readmission risk in pediatric patients with ED in our cohort. Isolated laboratory analyses and anthropometric measures showed limited predictive value in this context.

## 1. Introduction

Eating disorders (EDs) comprise a group of severe mental illnesses characterized by persistent disturbances in eating behavior and body image perception, with significant medical, nutritional, psychological, and social consequences. In pediatric and adolescent populations, EDs represent a major public health concern due to their high morbidity, potentially chronic course, and the frequent need for admission and readmission, even after apparent weight restoration [1,2,3,4].

Although the prognosis of EDs in children and adolescents is generally more favorable than in adults, a substantial proportion of patients experience relapse or a protracted disease course over the medium and long term [4,5]. Reported rates of complete remission vary widely due to heterogeneity in study design and follow-up duration, particularly in pediatric cohorts; nevertheless, progression to chronicity remains high, affecting approximately 20–40% of patients [4,6,7]. In this context, readmission emerges as a frequent and clinically relevant outcome, with a negative impact on both patients’ psychosocial development and healthcare systems. Moreover, outcomes such as readmission and length of hospital stay represent key indicators of healthcare utilization and resource burden, underscoring the importance of identifying factors associated with these events to optimize clinical management and improve the efficiency of care delivery.

Several studies have investigated factors associated with the risk of hospital readmission in patients with EDs. The most consistently reported predictors include lower weight gain during the initial hospitalization, low body mass index (BMI) at discharge, greater psychopathological severity—particularly in the presence of comorbid depression—and lower socioeconomic status [1,8,9,10]. However, most of these studies have primarily focused on clinical and psychological variables, with limited attention to a comprehensive and integrated nutritional characterization.

From a nutritional perspective, the assessment of malnutrition in EDs has traditionally relied on anthropometric parameters such as BMI or percentage of expected body weight. Nevertheless, these isolated measures may not adequately capture the complexity of nutritional and metabolic impairment in these patients, particularly during refeeding and partial recovery phases. In recent years, several biochemical and hormonal markers—including prealbumin, leptin, and the IGF-1/IGFBP-3 axis—have been proposed as complementary indicators of nutritional and metabolic status in EDs. However, their clinical utility as predictors of disease course and rehospitalization remains unclear and has shown inconsistent results in previous studies [11,12,13,14]. In this context, re-examining these markers in a well-characterized pediatric cohort may provide additional insight into their potential role in routine clinical practice, particularly when considered alongside anthropometric and clinical variables.

In addition, there is considerable heterogeneity in the response to nutritional rehabilitation during hospitalization, especially among patients presenting with moderate to severe malnutrition at diagnosis. Determining the degree of nutritional recovery that is clinically meaningful in reducing the risk of readmission remains an area of uncertainty, with limited evidence available in pediatric populations.

In this context, the primary aim of the present study was to identify nutritional factors—analytical, anthropometric, clinical, and evolutionary—associated with an increased risk of rehospitalization in pediatric patients with EDs admitted to a tertiary referral unit, as well as to determine the main variables associated with prolonged hospital stay. By doing so, this study seeks to provide evidence to optimize nutritional intervention strategies during hospitalization and post-discharge follow-up.

## 2. Materials and Methods

### 2.1. Study Design and Population

This was a retrospective observational study conducted at a tertiary referral hospital for patients diagnosed with eating disorders, covering the period from December 2022 to April 2025.

#### 2.1.1. Inclusion Criteria

- Age ≤ 18 years.
- Diagnosis of an eating disorder according to the Diagnostic and Statistical Manual of Mental Disorders, Fifth Edition (DSM-5) criteria.
- Fulfillment of the hospital admission criteria established in the institutional protocol for the management of hospitalized patients with eating disorders under 18 years of age (Appendix A).
- One or more hospitalizations in the pediatric and/or child and adolescent psychiatry wards of the study hospital.
- Post-discharge follow-up at the Eating Disorders Unit (EDU), defined as at least one outpatient visit following hospitalization (at least one month).

#### 2.1.2. Exclusion Criteria

- Diagnosis of avoidant/restrictive food intake disorder (ARFID).

#### 2.1.3. Sample Size

The sample size was determined by convenience, including all patients who met the inclusion criteria between December 2022 and April 2025. No a priori sample size calculation was performed due to the exploratory nature of the study and the lack of previous local data that would allow estimation of the effect size for the variables of interest.

### 2.2. Variables and Study Population Classification

Anthropometric measurements were collected at both admission and discharge by trained professionals using standardized measurement techniques. Weight and height were recorded for body mass index (BMI) calculation, along with triceps, biceps, subscapular, and suprailiac skinfolds, as well as mid-upper arm and waist circumferences. In addition to the absolute values, standard deviation scores (z-scores) for age- and sex-matched pediatric reference populations were calculated [15]. The Waterlow Index was also computed as an indicator of nutritional status, expressed as a percentage, both at admission and discharge. This index, originally described by John C. Waterlow [16], is based on weight-for-height and height-for-age relationships to distinguish acute (wasting) from chronic (stunting) malnutrition. In this study, it was calculated as (current weight/P50 weight for the corresponding height in the reference population) × 100, allowing the assessment of acute malnutrition.

Laboratory variables were obtained at admission and included markers commonly used in the literature to assess malnutrition: albumin (g/dL), prealbumin (mg/dL), ferritin (ng/mL), transferrin (mg/dL), retinol-binding protein (mg/dL), total cholesterol (mg/dL), AST (U/L), ALT (U/L), leptin (ng/mL), creatinine (mg/dL), total proteins (g/dL), vitamin D (ng/mL), alkaline phosphatase (U/L), T3 (ng/mL), T4 (ng/mL), TSH (mU/L), LH (mU/mL), FSH (mU/mL), IGF-1 (ng/mL), IGFBP-3 (mcg/mL), leukocyte count (mil/mm^3^), hemoglobin (g/dL), and hematocrit (%).

Clinical variables included sex, eating disorder subtype according to DSM-5 criteria, the presence of relevant medical comorbidities, bradycardia, hypotension, amenorrhea, self-harming or purging behaviors, and competitive exercise practice. Patients were included at any hospital admission for eating disorders, including both first admissions and readmissions. A history of previous hospitalizations for eating disorders was recorded and taken into account in the analysis, particularly for the evaluation of readmission outcomes. Because almost all patients had anorexia nervosa, stratified analyses by diagnosis were not feasible.

Evolutionary variables included length of hospital stay (days), duration of secondary amenorrhea (months) if present, time from diagnosis of the eating disorder to admission (months), highest recorded weight in the patient’s history, weight loss at admission relative to the highest recorded weight, and recovery of the Waterlow Index, expressed as the difference in percentage from admission to discharge.

Therapeutic variables included the need for nasogastric tube feeding, nutritional supplements, and treatment with vitamin D or thiamine during hospitalization.

The primary outcome variable was hospital readmission during the study period, defined as any new admission to the same hospital following discharge from the index hospitalization, regardless of clinical cause, provided it was related to the eating disorder diagnosis. A secondary outcome was length of hospital stay, expressed in days. Additionally, patients were stratified at admission according to the Waterlow Index, establishing subgroups based on the degree of malnutrition: mild (80–89%), moderate (70–79%), and severe (<70%).

### 2.3. Bias

Given the retrospective observational design of the study, the results may be subject to selection bias, as patients were recruited from a single tertiary hospital and specialized unit, as well as information bias due to the use of clinical records. Additionally, residual confounding from variables not collected or not recorded in the medical history cannot be ruled out.

### 2.4. Statistical Analysis

Statistical analyses were performed using IBM SPSS Statistics, version 30.

First, a descriptive analysis of the sample was conducted. For quantitative variables, measures of central tendency and dispersion were calculated, specifically the mean and standard deviation. Qualitative variables were described using absolute frequency (number of cases) and percentages.

To explore potential statistical differences between groups based on the presence or absence of hospital readmission during the study period, hypothesis testing was performed. For comparisons between quantitative variables and dichotomous qualitative variables, an independent samples Student’s t-test was used. Comparisons between two qualitative variables were analyzed using the chi-square (χ^2^) test or Fisher’s exact test when the sample size was small (expected frequency in one or more cells < 5). Prior to performing parametric tests, assumptions were assessed. Homogeneity of variances between groups was evaluated using Levene’s test. The distribution of quantitative variables was examined by visual inspection (histograms). Given the sample size, parametric tests were considered appropriate, as they are generally robust to moderate deviations from normality.

Finally, a multiple linear regression model was implemented using a backward stepwise variable selection method, with length of hospital stay (days) as the dependent variable and independent variables considered potentially influencing the duration of hospitalization. Given the limited number of events, the number of variables included in the regression model was restricted to reduce the risk of overfitting. Candidate variables were selected based on clinical relevance reported in the literature to ensure inclusion of the most meaningful predictors without compromising the sample size. A backward stepwise selection procedure was then applied to identify the most relevant predictors. This approach allows identification of qualitative variables that, having shown statistical significance in bivariate analysis, maintain an independent effect on the probability of readmission when adjusting for other covariates in the model. In this way, the study aimed to establish which factors acted as significant predictors of hospital stay duration in the analyzed population.

A *p*-value < 0.05 was considered statistically significant for all analyses described above.

### 2.5. Ethical Considerations

No personal identifying information was collected; patients were identified using a numerical code according to the order of admission. Confidentiality of patient data was strictly maintained. Original data are securely stored in the Pediatric and Child & Adolescent Psychiatry Service and are accessible only to the study investigators.

The study was conducted in accordance with the Declaration of Helsinki. The protocol was reviewed and approved by the Aragón Clinical Research Ethics Committee (CEICA).

## 3. Results

During the study period, a total of 79 patients with a diagnosis of eating disorders who required hospitalization were initially assessed. Of these, four were excluded for not meeting the inclusion criteria (diagnosis of ARFID), resulting in a final sample of 75 patients included in the analysis. No losses to follow-up were recorded, given the retrospective design of the study and the use of complete clinical records.

Biochemical, hormonal, and hematological parameters assessed at hospital admission are summarized in Table 1, including a descriptive analysis of the total sample and a comparison between patients who did not experience readmission and those who were readmitted during the follow-up period. When comparing both groups, readmitted patients tended to show lower nutritional markers and higher inflammatory and hepatic parameters compared with non-readmitted patients, while lipid levels were similar between groups. Overall, although trends toward less favorable nutritional and metabolic marker profiles were identified in patients who were readmitted, these differences did not reach statistical significance, likely due to the high intra-group variability of the analytical parameters.

The mean age of the sample was 14.5 years, with no relevant differences between patients who experienced readmission and those who did not. Table 2 summarizes the analysis of anthropometric variables. At admission, the mean body mass index (BMI) was 16.2 kg/m^2^, with slightly lower values observed in the readmission group compared with the non-readmission group. Similarly, a non-significant trend toward lower BMI Z-scores was observed among patients who were subsequently readmitted (−1.95 vs. −1.71).

During hospitalization, an overall increase in BMI was observed across the entire cohort, reaching a mean value of 17.5 kg/m^2^ at discharge. Both BMI and BMI Z-scores increased similarly from admission to discharge in both groups, with a mean BMI gain of 1.40 kg/m^2^ and a mean Z-score improvement of approximately 0.7 standard deviations.

Anthropometric measures related to skinfold thickness (tricipital, bicipital, subscapular, and suprailiac) were generally lower at admission in the readmission group compared with patients without readmission, although differences did not reach statistical significance. Arm circumference z-score (−2.21, *p* < 0.05) and waist circumference (55.47 cm) and z-score (−2.16) at admission were significantly lower in the readmission group. During hospitalization, all skinfolds and circumferences increased in both groups, with a generalized improvement in Z-scores. The increase in subscapular skinfold at discharge in the readmission group was statistically significant (7.34 cm, *p* < 0.05). Overall, the magnitude of change from admission to discharge was similar between groups, although trends toward slightly greater increases in tricipital skinfold and waist circumference were observed in the readmission group. Mid-upper arm and waist circumference also increased across the overall sample.

The Waterlow index was lower at admission in the readmission group (75.4 vs. 79.5) and increased at discharge in both groups, although these differences were not statistically significant. The mean change in the Waterlow index was 5.6 points in the total sample and was slightly greater among patients who experienced readmission.

Overall, these results indicate an improvement in anthropometric parameters from admission to hospital discharge across the entire cohort, both in patients with and without subsequent readmission. Baseline anthropometric status was less favorable among patients who were later readmitted; however, most differences did not reach statistical significance, likely due to the high variability of the analyzed anthropometric variables.

Regarding clinical, evolutionary, and treatment-related variables (Table 3, Table 4 and Table 5), none showed statistical significance as either a risk factor or protective factor for hospital readmission.

No statistically significant differences were observed in readmission rates according to the percentage of recovery of the Waterlow index in the total sample. However, when selecting patients with moderate to severe malnutrition at admission, a significant association was identified between a Waterlow index recovery below 5% and hospital readmission. In this subgroup, patients with <5% recovery had a significantly higher risk of readmission (OR 21; 95% CI: 2.19–200.8; *p* = 0.003). Recovery below 10% showed a trend toward increased readmission risk, although it did not reach statistical significance (*p* = 0.095). When considering only patients with severe malnutrition at admission, the sample size decreased considerably to 23 patients. In this subgroup, only one patient with >10% recovery was readmitted compared to six patients with <10% recovery. Despite this, the difference did not reach statistical significance (Table 6).

In the stepwise backward multiple regression model, with length of hospital stay as the dependent variable, all variables shown were initially entered into the model based on clinical relevance, and a backward stepwise procedure was applied. The use of nasogastric tube feeding was significantly associated with a reduction in hospital stay duration (B = −56.85; standardized β = −0.50; *p* < 0.001), representing the strongest predictor in the model. Additionally, the use of nutritional supplements was associated with a shorter hospitalization (B = −16.06; β = −0.30; *p* = 0.004), whereas a higher percentage of recovery according to the Waterlow index was related to longer hospital stays (B = 0.55; β = 0.23; *p* = 0.019). Furthermore, a higher number of previous admissions was associated with increased length of stay (B = 19.14; β = 0.24; *p* = 0.016). All other variables evaluated were sequentially removed during the stepwise procedure due to lack of statistical significance (Table 7).

## 4. Discussion

This study aimed to examine nutritional factors influencing hospital length of stay and risk of readmission in patients with an eating disorder. Unlike most previous research, which has focused mainly on weight recovery, our study integrates analytical, hormonal, and anthropometric variables as potential predictors of both readmission and length of stay.

Hospital readmission in patients diagnosed with an eating disorder is common and may be increasing, which entails a high economic and developmental cost during a critical stage of childhood [10,17]. Nevertheless, there is limited scientific evidence analyzing readmission as an outcome. In 2008, a study conducted by Hans-Christoph and colleagues reported that 45% of 212 adolescents with anorexia nervosa experienced at least one readmission [9]; in 2021, Marzola’s group published similar figures, with 39.4% of 170 patients with anorexia nervosa being readmitted [10]. Additionally, certain cohorts have described that up to 25% of patients with complete weight recovery required subsequent readmissions [1]. In our sample, 28% of patients (n = 21) were readmitted during the study period, and 16% (n = 12) had at least one previous admission, indicating that this is a frequent event that requires further investigation. Possible triggering factors for readmission include lower weight gain during hospitalization, younger age, and associated depressive symptoms, among others [1,9,10].

In the analysis of commonly reported laboratory variables related to nutritional status [18,19,20], comparisons between patients who required readmission and those who did not suggested a trend toward a more compromised nutritional and endocrine profile in the readmission group. First, prealbumin levels were, on average, lower in patients who were subsequently readmitted. Prealbumin is a sensitive marker of protein reserves responding to short-term nutritional changes, and its decrease has been associated with more severe malnutrition in patients with eating disorders and other states of starvation [14]. Elevated ferritin in readmitted patients could correlate with an inflammatory response or iron redistribution, although its interpretation is complex in eating disorders due to interactions between nutritional deficiency and inflammatory processes [21]. Endocrine markers also showed notable differences. For example, leptin levels were lower in readmitted patients. Recent evidence from a meta-analysis adjusted for BMI indicates that leptin levels are significantly altered in patients with eating disorders, even independently of BMI, being particularly low in anorexia nervosa, supporting leptin’s potential as a biomarker associated with disease severity and prognosis in these disorders [11]. IGF-1 and IGFBP-3 levels were also lower in the readmission group, consistent with studies showing that IGF-1 decreases with malnutrition and may reflect both nutritional severity and the response to caloric reintroduction during treatment [12,13].

In summary, the findings suggest that certain metabolic and hormonal biomarkers could help identify subgroups of patients with eating disorders who present greater metabolic compromise, supporting the notion that a broad and targeted laboratory assessment may contribute to improved risk stratification and individualization of clinical follow-up. However, these markers should not be interpreted as direct causal predictors of readmission. One important limitation of this study is that the available laboratory measurements were obtained at the time of hospital admission, a point at which most patients meet similar nutritional and metabolic criteria for hospitalization, potentially reducing the ability to detect differences between groups.

Beyond analytical findings, this study provides relevant data regarding anthropometric measurements. Consistent with previously published research, malnutrition based solely on anthropometric measures at admission was not associated with a higher risk of readmission [22]. Moreover, in our sample, despite improvement in most anthropometric measures and their z-scores at discharge, no association with increased readmission risk was observed. These findings underscore the need to develop indices that correlate anthropometric measures with other variables to identify malnutrition states that may be associated with slower recovery, with the aim of detecting this patient group and potentially allowing earlier intervention. In this context, more research groups are advocating for the systematic use of nutritional risk screening tools, such as STRONGkids and the Pediatric Nutrition Screening Tool (PNST), alongside anthropometric measurements in all pediatric patients at hospital admission [22,23,24,25]. Nevertheless, these tools have been criticized for low specificity in certain situations [26,27]. In patients with eating disorders, their utility is limited, as they tend to identify nearly all patients as at nutritional risk, reducing their discriminative value in this population.

One factor that demonstrated an influence on rehospitalization in our study was the degree of nutritional recovery achieved during hospitalization, measured by the percentage change in the Waterlow index, particularly in patients with moderate or severe malnutrition at admission. The Waterlow Index has been widely used to assess nutritional status in pediatric populations and remains a practical tool in hospitalized children at risk of disease-related malnutrition, as reflected in multicenter studies such as the DHOSPE study, which highlighted the high prevalence of malnutrition in pediatric inpatients and the utility of anthropometric indicators in routine clinical practice [28].

In the overall sample, no statistically significant differences in readmission rates were observed based on the percentage of Waterlow index recovery. However, when patients were stratified by nutritional severity at admission, clinically relevant differences emerged. In our study, the association between a Waterlow index recovery below 5% and an increased risk of readmission was observed only in the subgroup of patients with moderate to severe malnutrition at admission. This finding suggests that, in patients with greater baseline nutritional compromise, insufficient nutritional recovery during hospitalization may reflect incomplete stabilization, which could be associated with greater clinical vulnerability after discharge and, consequently, a higher likelihood of readmission. Conversely, using a 10% recovery threshold showed only a trend toward increased risk of rehospitalization in patients below this value, without reaching statistical significance. Moreover, the magnitude of the association observed for the 5% threshold should be interpreted with caution, as the estimated odds ratio was accompanied by a wide confidence interval (OR 21; 95% CI 2.19–200.8), indicating limited precision, likely due to the small sample size and the low number of events. Despite its utility, the Waterlow Index has inherent limitations, including relatively low specificity compared with more comprehensive nutritional assessment tools, potential inter-observer variability in anthropometric measurements, and lack of information on body composition or fluid shifts that may affect weight-based indicators. Therefore, while it remains a practical and widely accepted method for monitoring nutritional status, its results should be interpreted alongside clinical assessment and complementary nutritional parameters. Overall, these findings arise from subgroup analyses and should be considered exploratory rather than confirmatory. Further studies with larger sample sizes are needed to validate this association.

In our population, the main factor associated with reduced length of stay (days) was the use of a nasogastric tube (standardized B coefficient = −0.500; *p* < 0.001). Several hypotheses may explain this association. First, early enteral nutrition may facilitate adequate caloric intake from the initial stages of hospitalization, potentially reducing delays in refeeding due to refusal of oral intake, which is common in patients with eating disorders. Recent studies have shown that structured refeeding protocols, including early use of NG tubes when oral intake is insufficient, are associated with faster weight recovery and shorter hospital stays, without increased risk of refeeding syndrome [29,30,31]. Similarly, the use of oral nutritional supplements was associated with a significant reduction in length of stay (standardized B = −0.295; *p* = 0.004). These findings suggest that both the use of NG tubes when clinically indicated and the administration of nutritional supplements may be associated with reduced variability in energy intake and facilitate achieving nutritional goals, allowing earlier attainment of clinical stability criteria necessary for discharge, thereby underscoring the importance of nutritional support in reducing length of hospital stay. However, the association between nasogastric tube use and shorter length of stay should be interpreted cautiously, as this intervention is not randomly assigned and may be influenced by baseline severity (confounding by indication). Therefore, residual confounding cannot be excluded.

A history of previous hospitalizations was independently associated with longer hospital stays (standardized B = 0.242; *p* = 0.016), indicating that patients with multiple hospitalizations often have more chronic disease, greater treatment resistance, and slower weight recovery, leading to prolonged hospitalizations. Likewise, the Waterlow recovery index showed a positive association with length of stay (standardized B = 0.231; *p* = 0.019). This result suggests that greater malnutrition at admission, reflected by a lower Waterlow index, leads to longer nutritional recovery and, therefore, longer hospitalization. However, this relationship may be bidirectional: a longer hospital stay could also allow for greater nutritional recovery, rather than recovery solely predicting length of stay.

In contrast, other clinical and analytical variables—including medical comorbidities, bradycardia, hypotension, purging behaviors, excessive exercise, disease duration, maximum previous weight, or weight loss—were not retained in the final model. This suggests that, although these variables are clinically relevant, their effect on length of stay may be mediated by overall nutritional severity and treatment response during hospitalization.

## 5. Strengths and Limitations

Among the strengths of this study is, first, a well-characterized clinical cohort from a specialized reference unit, ensuring homogeneous patient management according to standardized care protocols and routine clinical practice. This reduces therapeutic variability and reinforces the internal validity of the results. Second, the study includes comprehensive and detailed data collection at both admission and discharge, obtained by trained professionals using standardized techniques, allowing simultaneous integration of anthropometric, analytical, clinical, evolutionary, and therapeutic variables.

Limitations include the moderate sample size, particularly the limited number of readmission events (n = 21), which may affect the stability and reliability of multivariable models. This increases the risk of overfitting and limits the robustness of the estimated associations. Therefore, the results of regression analyses should be interpreted with caution. This study should be interpreted as hypothesis-generating, given its retrospective design and limited sample size. Some analyzed variables showed high internal variability, reducing the power to detect statistically significant differences. Another important limitation is the absence of a multivariable logistic regression model specifically addressing hospital readmission. Although this was partly due to the limited sample size as well as the relatively high number of potential predictor variables, this restricts the ability to identify independent predictors of readmission and to control for potential confounding factors. Therefore, the associations observed should be interpreted with caution. Finally, as this cohort comes from a single hospital center, generalization of results should be approached with caution.

## 6. Conclusions

Nutritional support via nasogastric tube when indicated and the use of nutritional supplements were associated with shorter hospital stays in pediatric patients with ED in our cohort. In addition, among patients with moderate to severe malnutrition, a Waterlow index recovery below 5% was associated with a higher probability of readmission; however, this finding should be interpreted with caution given its exploratory nature. Isolated laboratory analyses and anthropometric measures showed limited predictive value in this context. These findings support the potential relevance of intensive nutritional rehabilitation strategies during hospitalization, although further studies are needed to confirm these associations.

## Figures and Tables

**Table 1:** Laboratory variables of patients with eating disorders treated at our hospital between 2022 and 2025: comparison between patients with readmission and no readmission.

	Total Average (SD)	No Readmission (54) Average (SD)	Readmission (21) Average (SD)
Albumin (g/dL)	4.72 (2.34)	4.73 (2.78)	4.69 (0.21)
Prealbumin (mg/dL)	23.60 (22.69)	24.74 (6.42)	20.63 (6.01)
Total proteins (g/dL)	7.26 (0.69)	7.11 (0.74)	7.64 (0.34) ^1^
Ferritin (ng/mL)	172.1 (100.7)	158.5 (97.28)	208.1 (103.3)
Transferrin (mg/dL)	214.5 (63.19)	217.8 (70.19)	205.7 (40.17)
Retinol-binding protein (mg/dL)	3.51 (0.91)	3.59 (0.91)	3.30 (0.91)
Total cholesterol (mg/dL)	198 (58.51)	196.8 (61.26)	201.1 (51.85)
AST (U/L)	25.01 (10.36)	24.19 (10.84)	27.32 (8.74)
ALT (U/L)	23.52 (21.56)	20.60 (18.45)	31.25 (27.24)
Leptin (ng/mL)	5.21 (12.09)	5.93 (14.10)	3.31 (2.58)
Creatinine (mg/dL)	0.78 (0.17)	0.78 (0.17)	0.78 (0.14)
Vitamin D (ng/mL)	28.41 (8.52)	28.52 (8.82)	28.12 (7.98)
Alkaline phosphatase (U/L)	89.80 (44.64)	89.29 (44.54)	91.16 (46.10)
T3 (ng/mL)	2.01 (0.70)	2.00 (0.73)	2.02 (0.64)
T4 (ng/mL)	1.05 (0.16)	1.04 (0.13)	1.06 (0.21)
LH (mU/mL)	3.37 (5.05)	3.56 (4.99)	2.89 (5.31)
FSH (mU/mL)	3.27 (2.53)	3.07 (2.37)	3.77 (2.91)
IGF-1 (ng/mL)	145.4 (89.72)	155.8 (92.17)	119.2 (79.48)
IGFBP-3 (mcg/mL)	5.36 (4.42)	5.78 (5.10)	4.33 (1.46)
Leukocites (mil/mm^3^)	5.63 (1.63)	5.73 (1.68)	5.36 (1.48)
Hemoglobin (g/dL)	13.72 (1.09)	13.67 (1.23)	13.87 (0.58)
Hematocrit (%)	40.25 (3.05)	40.05 (3.30)	40.78 (2.21)

^1^ *p*: differences from the no readmission group (*p* < 0.05). SD: standard deviation. AST: Aspartate aminotransferase. ALT: Aspartate aminotransferase. T3: triiodothyronine. T4: thyroxine. LH: Luteinizing hormone. FSH: Follicle-stimulating hormone. IGF-1: Insulin-like growth factor 1. IGFBP-3: IGF-1 binding protein 3.

**Table 2:** Anthropometric variables of patients with eating disorders treated at our hospital between 2022 and 2025: comparison between patients with readmission and no readmission.

	At Admission			At Discharge			Change from Admission to Discharge		
	Total Average (SD)	No Readmission Average (SD)	Readmission Average (SD)	Total Average (SD)	No Readmission Average (SD)	Readmission Average (SD)	Total Average (SD)	No Readmission Average (SD)	Readmission Average (SD)
Age (years)	14.54 (1.99)	14.69 (2.08)	14.15 (1.72)	-	-	-	-	-	-
BMI (kg/m^2^)	16.20 (3.09)	16.36 (3.23)	15.78 (2.71)	17.49 (2.58)	17.62 (2.60)	17.18 (2.57)	1.40 (0.93)	1.40 (0.94)	1.40 (0.91)
BMI (z-score)	−1.77 (1.37)	−1.71 (1.47)	−1.95 (1.09)	−1.11 (1.02)	−1.06 (1.10)	−1.22 (0.81)	0.69 (0.68)	0.67 (0.72)	0.73 (0.59)
Triceps skinfold (cm)	9.18 (5.08)	9.49 (5.30)	8.36 (4.50)	10.12 (3.96)	10.37 (4.13)	9.41 (3.46)	1.75 (2.66)	1.61 (2.59)	2.13 (2.88)
Triceps skinfold (z-score)	−1.45 (1.13)	−1.39 (1.20)	−1.61 (0.92)	−1.23 (0.91)	−1.17 (0.93)	−1.38 (0.87)	0.39 (0.63)	0.37 (0.64)	0.42 (0.63)
Biceps skinfold (cm)	5.92 (3.57)	6.29 (3.71)	4.87 (2.98)	6.61 (3.07)	6.87 (3.14)	5.91 (2.82)	1.02 (2.78)	1.01 (2.66)	1.05 (3.21)
Biceps skinfold (z-score)	−1.21 (1.20)	−1.09 (1.22)	−1.55 (1.12)	−0.93 (1.07)	−0.84 (1.09)	−1.18 (1.00)	0.43 (0.93)	0.45 (0.84)	0.39 (1.17)
Subscapular skinfold (cm)	7.43 (4.82)	7.94 (5.15)	6.08 (3.59)	8.78 (3.49)	9.30 (3.54)	7.34 (2.97) ^1^	2.08 (3.05)	2.07 (3.38)	2.09 (1.99)
Subscapular skinfold (z-score)	−1.29 (1.25)	−1.16 (1.32)	−1.64 (0.99)	−0.94 (0.93)	−0.82 (0.88)	−1.27 (1.00)	0.56 (0.66)	0.57 (0.73)	0.55 (0.42)
Suprailiac skinfold (cm)	7.80 (5.46)	8.13 (5.66)	6.84 (4.89)	9.41 (4.76)	9.85 (4.92)	8.17 (4.17)	2.39 (4.14)	2.46 (4.20)	2.19 (4.09)
Suprailiac skinfold (z-score)	−0.69 (4.14)	−0.44 (4.75)	−1.40 (1.27)	−0.65 (1.16)	−0.53 (1.15)	−0.98 (1.16)	0.22 (3.84)	0.08 (4.39)	0.63 (1.25)
Arm circumference (cm)	20.31 (2.99)	20.53 (3.18)	19.73 (2.40)	20.95 (2.37)	21.13 (2.51)	20.43 (1.88)	0.59 (3.62)	0.53 (3.94)	0.77 (2.67)
Arm circumference (z-score)	−1.73 (1.29)	−1.55 (1.41)	−2.21 (0.75) ^1^	−1.52 (0.92)	−1.46 (0.99)	−1.67 (0.70)	0.45 (0.56)	0.40 (0.56)	0.57 (0.56)
Waist circumference (cm)	58.05 (6.42)	59.02 (6.84)	55.47 (4.30) ^1^	60.78 (6.26)	61.39 (6.94)	59.18 (3.62)	2.58 (9.93)	1.90 (11.38)	4.44 (3.38)
Waist circumference (z-score)	−1.71 (1.35)	−1.54 (1.40)	−2.16 (1.12) ^1^	−0.77 (1.05)	−0.78 (0.89)	−0.75 (1.41)	1.12 (1.25)	0.95 (0.80)	1.58 (1.99)
Waterlow Index (%)	78.40 (13.96)	79.52 (15.21)	75.37 (9.51)	83.50 (14.19)	84.19 (15.72)	81.68 (9.02)	5.60 (11.31)	5.11 (12.92)	6.99 (4.55)

^1^*p*: differences from the no readmission group (*p* < 0.05). SD: standard deviation. BMI: Body mass index.

**Table 3:** Clinic variables of patients with eating disorders treated at our hospital between 2022 and 2025: comparison between patients with readmission and no readmission.

	Total (*n* = 75)	No Readmission (*n* = 54)	Readmission (*n* = 21)
Sex	Women: 94.7% (71) Men: 5.3% (4)	Women: 92.6% (50) Men: 7.4% (4)	Women: 100% (21) Men: 0
Eating Disorder Diagnosis	Anorexia nervosa: 98.7% (74) Bulimia nervosa: 1.3% (1)	Anorexia nervosa: 98.1% (53) Bulimia nervosa: 1.9% (1)	Anorexia nervosa: 100% (21) Bulimia nervosa: 0
Medical Comorbidities	34.7% (26)	40.7% (22)	19% (4)
Bradycardia	32.0% (24)	33.3% (18)	28.6% (6)
Hypotension	36.0% (27)	29.6% (16)	52.4% (11)
Amenorrhea	53.3% (40)	46.3% (25)	71.4% (15)
Self-harm	37.3% (28)	33.3% (18)	47.6% (10)
Purging Behaviors	29.3% (22)	31.5% (17)	23.8% (5)
Competitive exercise	45.3% (34)	38.9% (21)	61.9% (13)
Previous admissions	16.0% (12)	14.8% (8)	19% (4)

Data are expressed as a percentage (*n*).

**Table 4:** Evolutive variables of patients with eating disorders treated at our hospital between 2022 and 2025: comparison between patients with readmission and no readmission.

	Total Average (SD)	No Readmission Average (SD)	Readmission Average (SD)
Days of Admission	40.37 (26.83)	39.07 (26.39)	43.71 (28.31)
Duration of Secondary Amenorrhea (months)	6.33 (6.74)	7.00 (7.83)	5.20 (4.41)
Duration of Illness (months)	10.48 (10.17)	11.22 (10.45)	8.57 (9.38)
Maximum Recorded Weight (kg)	50.04 (12.55)	50.23 (12.92)	49.57 (11.86)
Weight Loss (kg)	−8.75 (7.61)	−8.87 (8.02)	−8.44 (6.66)
Waterlow % Recovery	5.60 (11.31)	5.11 (12.92)	6.99 (4.55)

SD: standard deviation. Weight loss: Maximum Recorded Weight—Admission Weight.

**Table 5:** Treatment variables of patients with eating disorders treated at our hospital between 2022 and 2025: comparison between patients with readmission and no readmission.

	Total (*n* = 75)	No Readmission (*n* = 54)	Readmission (*n* = 21)
Nasogastric tube feeding	8.0% (6)	7.4% (4)	9.5% (2)
Oral nutritional supplements	52.0% (39)	55.6% (30)	42.9% (9)
Vitamin D treatment	24.0% (18)	25.9% (14)	19% (4)
Thiamine treatment in patients at risk of refeeding syndrome	44.0% (33)	46.3% (25)	38.1% (8)

Data are expressed as a percentage (*n*).

**Table 6:** Association between percentage of Waterlow index recovery and hospital readmission according to nutritional status at admission.

Total (*n* = 68)				
		**Readmission** **%** ** (*n*)**	**No Readmission** **%** ** (*n*)**	***p* Value; OR (95% CI)**
**Waterlow recovery**	<5%	10.3% (7)	26.5% (18)	0.827; OR 1.1 (0.37–3.43)
	>5%	16.2% (11)	47.1% (32)	
	<10%	22.1% (15)	55.9% (38)	0.520; OR 1.57 (0.39–6.39)
	>10%	4.4% (3)	17.6% (12)	
**Moderate and Severe Malnutrition at Admission According to Waterlow Index (** ***n*** ** = 43)**				
		**Readmission** **%** **(*n*)**	**No Readmission** **% (** ***n*** **)**	***p*** ** Value; OR (95% CI)**
**Waterlow recovery**	<5%	14.0% (6)	2.3% (1)	0.003; OR 21 (2.19–200.8)
	>5%	18.6% (8)	65.1% (28)	
	<10%	27.9% (12)	39.5% (17)	0.095; OR 4.23 (0.79–22.48)
	>10%	4.7% (2)	27.9% (12)	
**Severe Malnutrition at Admission According to Waterlow Index (** ***n*** ** = 23)**				
		**Readmission** **% (** ***n*** **)**	**No Readmission** **% (** ***n*** **)**	***p*** ** Value; OR (95% CI)**
**Waterlow recovery**	<5%	8.7% (2)	4.3% (1)	0.209; OR 6.0 (0.4–87.5)
	>5%	21.7% (5)	65.2% (15)	
	<10%	26.1% (6)	34.8% (8)	0.176; OR 6.0 (0.58–61.8)
	>10%	4.3% (1)	34.8% (8)	

Data are expressed as a percentage (*n*). Waterlow recovery refers to the percentage of weight recovery calculated using the Waterlow index. *p* values < 0.05 were considered statistically significant.

**Table 7:** Stepwise Backward Multiple Linear Regression for Length of Hospital Stay.

	Coefficient B	Standardized Coefficient B	*p*
NG Tube	−56.853	−0.500	<0.001
Previous admissions	19.141	0.242	0.016
Nutritional Supplements	−16.059	−0.295	0.004
Waterlow recovery	0.551	0.231	0.019
Thiamine Treatment	−7.958	−0.146	0.221
Vitamin D Treatment	-	-	-
Medical Comorbidities	-	-	-
Bradycardia	-	-	-
Hypotension	-	-	-
Self-harm	-	-	-
Purging Behaviors	-	-	-
Exercise	-	-	-
Duration of Illness (months)	-	-	-
Maximum Recorded Weight	-	-	-
Weight Loss	-	-	-
Readmission	-	-	-

A stepwise backward multiple linear regression model was performed to identify factors associated with length of hospital stay. Unstandardized coefficients (B), standardized coefficients (β), and *p* values are reported. Variables not retained in the final model are indicated by dashes. Statistical significance was set at *p* < 0.05.

## Data Availability

The data extracted from the studies and any other material used in the review are presented in the different sections of the work, in the annexes, or referenced in the bibliography.

## Appendix A. Admission Criteria Established in the Institutional Protocol Management of Hospitalized Patients with Eating Disorders Under 18 Years of Age

**Nutritional criteria**

- Persistent weight loss: >25% of previous body weight within <6 months or >10% within 1 month.
- Body mass index (BMI) <15 kg/m^2^.
- BMI <16 kg/m^2^ in the presence of acute weight loss, medical complications, or refusal to eat.

**Medical criteria**

- Heart rate <50 beats per minute (<45 bpm at night).
- Blood pressure <80/50 mmHg and/or related symptoms.
- Orthostatic hypotension: changes >20 bpm or >10 mmHg.
- Electrolyte disturbances: potassium <3 mEq/L, sodium <130 mEq/L, and/or ECG abnormalities.
- Severe hypoproteinemia and/or peripheral edema.
- Persistent hypoglycemia and/or elevated transaminases.
- Dehydration or fluid-electrolyte imbalance.
- Hypothermia (temperature <35.5 °C).
- Hepatic, renal, or cardiovascular failure.
- Other acute complications (e.g., pancreatitis, gastrointestinal bleeding).

**Psychiatric criteria**

- Complete refusal to eat or inability to control bulimic behaviors (uncontrolled use of laxatives or diuretics).
- Uncontrollable hyperactivity.
- Failure of outpatient treatment.
- Pregnancy at risk of miscarriage.
- Geographic barriers or severe family conflicts.
- Suicide attempts or high-risk suicidal ideation.
- Associated psychiatric disorders
